# Safety tests and clinical research on buccal and nasal microneedle swabs for genomic analysis

**DOI:** 10.3389/fbioe.2023.1296832

**Published:** 2023-12-05

**Authors:** JeongHyeon Kim, Jae-Woo Moon, Gyeong Ryeong Kim, Wonsub Kim, Hae-Jin Hu, Won-Jun Jo, Seung-Ki Baek, Gil-Hwan Sung, Jung Ho Park, Jung-Hwan Park

**Affiliations:** ^1^ Department of Bionano Technology and Gachon BioNano Research Institute, Gachon University, Seongnam, Republic of Korea; ^2^ Endomics Inc, Seongnam, Republic of Korea; ^3^ QuadMedicine R&D Centre, QuadMedicine Co. Ltd., Seongnam, Republic of Korea; ^4^ Department of Medicine, Kangbuk Samsung Hospital, Sungkyunkwan University School of Medicine, Seoul, Republic of Korea

**Keywords:** buccal microneedle swab, nasal microneedle swab, genomic analysis, safety test, clinical research

## Abstract

Conventional swabs have been used as a non-invasive method to obtain samples for DNA analysis from the buccal and the nasal mucosa. However, swabs may not always collect pure enough genetic material. In this study, buccal and nasal microneedle swab is developed to improve the accuracy and reliability of genomic analysis. A cytotoxicity test, a skin sensitivity test, and a skin irritation test are conducted with microneedle swabs. Polymer microneedle swabs meet the safety requirements for clinical research and commercial use. When buccal and nasal microneedle swabs are used, the amount of genetic material obtained is greater than that from commercially available swabs, and DNA purity is also high. The comparatively short microneedle swab (250 μm long) cause almost no pain to all 25 participants. All participants also report that the microneedle swabs are very easy to use. When genotypes are compared at five SNP loci from blood of a participant and from that person’s buccal or nasal microneedle swab, the buccal and nasal microneedle swabs show 100% concordance for all five SNP genotypes. Microneedle swabs can be effectively used for genomic analysis and prevention through genomic analysis, so the utilization of microneedle swabs is expected to be high.

## 1 Introduction

Genetic analysis is a powerful tool to identify gene function and control, disease classification, biomarker identification and drug discovery (International, 2004; Chen et al., 2009). DNA sequence variation can occur within species, often in the form of single nucleotide polymorphisms (SNPs). SNPs are single base-pair changes that occur at specific positions along the DNA strand, representing genetic variations within a population. They can influence various traits, including susceptibility to diseases, physical attributes, and drug responses (Manolio et al., 2008; Chen et al., 2009).

There are two common methods of collecting DNA for genetic analysis: using a syringe to draw blood, and using a swab to retrieve samples of saliva or mucosal tissue (Walker et al., 1999; Lee and Ladd, 2001). Blood collection is commonly used because blood circulates throughout tissues and biomarkers such as DNA are abundant in blood (Yip et al., 2017; Donohue et al., 2019). However, this method has several disadvantages: significant pain caused by the syringe, difficulty in collecting blood from the elderly, the risk of infection, and the necessity of medical expertise (Vagnoli et al., 2015; Cheng et al., 2016; Fukuroku et al., 2016; Chen et al., 2022). Swabs, on the other hand, are easy use, cause less pain, and thus facilitate greater patient compliance (Walker et al., 1999; McBride et al., 2010). Samples of saliva and tissues separated from mucosal tissue can be collected easily, mainly from the buccal mucosa, by using a swab (Theda et al., 2018; Kam et al., 2020). However, swabs have the disadvantages of greater difficulty in collecting a sufficient amount of DNA and the resulting lower DNA purity (Lee and Ladd, 2001).

Microneedles are used to deliver drugs or to extract body fluid from the skin with little pain (Park et al., 2005). Microneedles have been reported to be clinically safe, although minor problems such as skin irritation and erythema have been reported (Chen et al., 2019; Santos et al., 2021). However, the pain caused by microneedles depends on the length of the microneedle (Gill et al., 2008; Jeong et al., 2017). Shorter microneedles with length less than 500 μm cause very little pain and skin was resealed within about 20 min after insertion (Bal et al., 2008; Haq et al., 2009; Gupta et al., 2011; Chen et al., 2019). Thus, shorter microneedles have been manufactured for commercial use in fields such as the cosmetics industry (Kim et al., 2012).

Currently, microneedles are used diagnostically to detect metabolites such as glucose and cholesterol or cell-free nucleic acids present in the interstitial fluid by absorbing interstitial fluid or by delivering interstitial fluid through the microchannel of hollow microneedles (Al Sulaiman et al., 2019). However, microneedles have not been used to directly collect tissue samples. In particular, microneedles have not been used to collect biomarkers from mucous membranes, including the oral and nasal cavity. In a previous study, we developed and optimized a polymeric microneedle swab for sampling buccal mucosa noninvasively. *Ex-vivo* and *in-vivo* studies resulted in the development of a microneedle swab that can retrieve a greater amount of DNA and also provide greater DNA purity compared to commercially available cotton swabs. In addition, animal experiments have shown that a microneedle swab enables the analysis of biomarkers in the buccal mucosa that contain genetic information (Kim et al., 2022).

In the current study, two types of microneedle swabs, buccal and nasal microneedle swab, were fabricated in established way from the results of previous studies. Clinical efficacy was observed in four areas when these microneedle swabs were applied to the buccal and nasal cavities: (International, 2004): medical grade buccal and nasal microneedle swabs were produced for safety and clinical research, (Chen et al., 2009), the amount and purity of DNA collected from buccal and nasal mucosa were evaluated, (Manolio et al., 2008), the genotypes of the five SNP loci in the obtained DNA were compared with the corresponding genotypes obtained from DNA in blood, and (Walker et al., 1999) the safety of a microneedle swab was evaluated. Through this clinical study, the safety and clinical efficacy of nasal and buccal microneedle swabs were verified.

## 2 Materials and methods

### 2.1 Characteristics of microneedle swab

Buccal and nasal microneedle swabs consist of a head and a handle connected to the head (Kim et al., 2022). Microneedles are on both sides of the head. There is a breakpoint on the handle so that the head can be separated and put into a 1.5-mL tube after the sample is obtained. The difference between microneedles for the buccal swab and for the nasal swab is the size of the head and the number of microneedles. However, the number of microneedles per unit area is the same for both swabs.

The buccal microneedle swab has a total of 890 microneedles, 445 per side. The nasal microneedle swab has a total of 502 microneedles, 251 on each side. In both swabs, microneedles are arranged in a zigzag pattern. The height of the microneedles is 250 μm, and the distance between the microneedles is 200 μm. The sharpness of the tip is 20 μm (Table 1A). The total length of the buccal microneedle swab is 14 cm, and the breakpoint of the handle is 3 cm below end of the head. The length and breakpoint of the nasal microneedle swab are 10.5 cm and 2.5 cm, respectively. The head of the buccal microneedle is 6 mm wide by 15 mm long by 2 mm thick. The head of the nasal microneedle swab is 5 mm wide by 10 mm long by 2 mm thick (Table 1B).

**TABLE 1:** Geometric characteristics. (A) Microneedles of buccal swab and nasal swab. (B) Geometric characteristics of commercial swabs (Isohelix, Noblebio) and buccal and nasal microneedle swabs.

(A) MN swab	Height [μm]	Number [ea]	Interval [μm]	Width [μm]	Sharpness [μm]
Buccal	250	890	200	200	20
Nasal	250	502	200	200	20

(B) Swab characteristics		Total	Head			Shaft	
		Length [cm]	Length [cm]	Width [cm]	Thickness [cm]	Length [cm]	Breakpoint [cm]
Buccal	Isohelix	16	1.5	0.75	0.3	14.5	4
	MN swab	14	1.5	0.6	0.2	12.5	3
Nasal	Noblebio	15	1.5	0.3	0.3	13.5	9
	MN swab	10.5	1	0.5	0.2	9.5	2.5

Polymeric microneedle swabs were manufactured by injection molding obtained from Cyclic Olefin Copolymer (COC) in Quad Medicine’s medical device GMP (good manufacturing practice) facility (Seongnam-si, Gyeonggi-do, Korea) (Supplementary Figure S1, S2). Polymeric microneedle swabs were prepared using injection molding. Molds for buccal swabs and nasal swabs were made of stainless steel using a computer numerical control (CNC) machine. The stainless steel mold can also be used to mold other thermoplastic polymers. The injection temperature was 200°C–250°C, and the injection pressure was 80–110 MPa. Commercial buccal swabs were SK-2S (rayon swabs) from Isohelix, and commercial nasal swabs were NFS-1 (flocked nylon swabs) from Noblebio. The geometries of microneedle swabs and commercial swabs were observed using a mirrorless camera (Canon EOS RP, Japan), a stereo microscope (Leica M205, Wetzlar, Germany), and a scanning electron microscope.

### 2.2 Safety tests of microneedle swab

Prior to clinical tests, cytotoxicity, skin sensitivity, and skin irritation tests were performed to confirm the safety of the microneedle swab.

#### 2.2.1 Cytotoxicity test

Mouse fibroblast NCTC clone 929 (L-929; American Type Culture Collection, United States) was used to evaluate the toxicity of the microneedle swab. The test substance was eluted using minimum essential medium (MEM; liquid, gibco) with 10% (v/v) horse serum (gibco) and 1% (v/v) penicillin-streptomycin (gibco). The elute was brought into direct contact with the cells, and the reaction was observed under a microscope. MEM medium containing no substance was used as a solvent control (Reagent control), and high-density polyethylene film and ZDEC polyurethane film (Hatano Research Institute, Food and Drug Safety Center, Japan) were used as negative and positive control materials, respectively. The head of the microneedle swab was broken at the breakpoint (6 cm^2^/1 mL 10% MEM medium), placed in the medium, and stirred for 24 h in a 37 °C 5% CO_2_ incubator. Negative and positive controls were eluted at a rate of 1 mL of 1× MEM medium per 0.1 g at 37 °C for 24 h in a 5% CO2 incubator with stirring. All eluates were not pH adjusted, stored at room temperature, and used within 4 h. The monolayer of cultured cells was treated with trypsin (trypsin/EDTA, gibco) to adjust the cell concentration to 10^5^ per 1 mL, and the cultured cells were injected (2 mL each) into about 10 cm^2^ wells (6-well tissue culture plate, Φ 35 mm/well). After culturing for more than 24 h in a 37 °C, 5% CO_2_ incubator, monolayer of cultured wells was selected, marked as either a test substance treatment group or a control group (a solvent control group, a negative control group, and a positive control group), and then the medium was removed. The test substance treatment group, a solvent control group, a negative control group, and a positive control group were dispensed into selected wells and incubated for 48 h in a 37 C 5% CO_2_ incubator. After culturing, cell lysis and morphology were observed under a microscope (Nikon, Japan). The presence of a uniform monolayer of cells was expressed as (+), and the absence of a confluent monolayer was expressed as (−). When the color of the medium changed to yellow after elution, it was determined that the medium was changed to acidic by the eluted material, and when it turned crimson or purple, it was determined that the medium was changed to basic. To demonstrate the validity of the test, the solvent control group and the negative control group should not show cytotoxicity (Grade 0), and the positive control group should show moderate to high cytotoxicity (> Grade 2).

#### 2.2.2 Skin sensitivity test

In order to evaluate the skin sensitivity of microneedle swabs, sensitization was induced by intradermal injection and topical application of polar and non-polar solvent eluates with 300–370 g Dunkin Hartley guinea pigs (Samtako Bio Korea, Gyeonggi-do, Korea). The mortality, general symptoms, and skin sensitivity of the guinea pigs were evaluated. This test was approved by the Animal Ethics Committee (approval number: IAC 2022-2242) and carried out in accordance with the Law on Laboratory Animals [Law No. 18969 (2020-06-10, partially amended)] and standard operating guidelines.

Sterile saline (Daihan Pharm. Co., Seoul, Korea) was used as a polar elution solvent and cottonseed oil (Junsei Chemical Co., Tokyo, Japan) was used as a non-polar elution solvent. The elution rate was 6 cm^2^/mL (surface area of the test substance: 4.88 cm^2^/ea) and eluted by stirring in a shaking water bath (50 °C, 72 h). The solvent control group was eluted under the same conditions. The test groups for each elution solvent (G2, G4) consisted of 10 guinea pigs each, and the control group (G1, G3) consisted of 5 guinea pigs each. Intradermal induction and local induction were carried out by shaving the intrascapular region, and induction and skin reaction evaluation were performed by shaving the upper flank.

In the intradermal induction step, three samples of solvent, solvent and Freund’s complete adjuvant (FCA; Sigma-Aldrich) (1:1), and solvent, FCA, and extract (1:1:1) were intradermally injected into the left and right sides of the skin of the scapula, respectively (Supplementary Figure S3A,B).

For the topical induction step, 0.5 mL of 10% sodium dodecyl sulfate (SDS; BIONEER) was applied 5 days after completion of intradermal induction. A filter paper (2 cm × 4 cm) was wetted with the test substance (0.4 mL) and applied to the intradermal injection site for 48 h The control substance (0.4 mL) was applied in the same manner.

In the induction phase, at 13 days after the local induction phase, filter papers (2 cm × 2 cm) wetted with the test substance (left, 0.2 mL) and the control substance (right, 0.2 mL) were placed for 24 h using Coban (TM, 3M) and then removed.

The skin reaction was evaluated according to the table (Supplementary Figure S3C). If the control group’s grade is less than 1 grade and the test group’s grade is 1 or higher, it is considered to indicate skin sensitization of the test group.

#### 2.2.3 Skin irritation test

The eluate from the microneedle swab was administered intradermally to NZWnl rabbits (Pizhou Dongfang Breeding, China) to evaluate skin irritation. This was approved by the Animal Ethics Committee (approval number: IAC 2022-2133) and performed in accordance with the Law on Laboratory Animals [Law No. 18969 (2020-06-10, partially amended)] and standard operating guidelines.

The head of the microneedle swab was separated at the breakpoint, and the head was put in sterile saline solution (Daihan Pharm. Co., Ltd) and cottonseed oil (Junsei Chemical Co., Ltd). The skin irritation test was performed in the same manner as the skin sensitivity test.

After the rabbits’ body weight was measured, hair was removed from the back before administration of the test substance, and three healthy rabbits weighing 2.0 kg or more with clean skin were selected. On the day of administration, 0.2 mL each of the extracts of sterile physiological saline and cottonseed oil and each solvent control material were administered intradermally to 5 sites near the rabbit’s spine (Supplementary Figure S4A).

Changes in general symptoms and the death of any animals were observed for all animals once a day for 3 days. Body weight change was measured three times. Immediately after administration, the presence of abnormalities at the administration site was checked, and erythema, crust formation, and degree of edema formation were observed and recorded according to grade at 24, 48, and 72 h after administration (Supplementary Figure S4B). Immediately after intradermal administration, photographs were taken to record the shape of the injection site.

Skin irritation was evaluated by adding up all grades of erythema observed at 24, 48, and 72 h after administration, and then dividing the total number of observations by 15 (3 scoring time points × 5 test injection sites). The values calculated for each animal were added together and divided by 3. When the difference between the scores of the test substance and the control substance was 1.0 or less, it was considered that there was no skin irritation.

### 2.3 Clinical test of microneedle swabs

#### 2.3.1 Sterilization of microneedle swabs

Sterilization and validation of sterilization of the microneedle swab were confirmed by Greenpia Technology Co., Ltd. (Yeoju-si, Gyeonggi-do, Korea). Buccal and nasal microneedle swabs were treated with 15.0 kGy gamma sterilization for clinical study. Sterility validation was performed according to the validation protocol described in ISO11137-2:2013.

#### 2.3.2 Clinical research and subjects

The clinical research was conducted by randomly recruiting 25 healthy subjects from Kangbuk Samsung Hospital. The participants were between 19 and 70 years old, and informed consent was obtained from all participants. This study was approved by the Kangbuk Samsung Hospital Clinical Review Board (IRB File No: KBSMC 2022-10-001-001).

#### 2.3.3 Sampling method

Commercial swabs were used as controls. A commercially available rayon swab (SK-2S, Isohelx) was used for the buccal swab, and a flocked nylon swab (NFS-1, Noblebio) was used for the nasal swab.

SK-2S buccal swab sampling was carried out according to the manufacturer’s instructions. For buccal microneedle swab, buccal samples were collected by rubbing each side of the swab 5 times for a total of 10 times of wiping both sides (Supplementary Figure S5A). Samples were collected by rubbing the inside of the cheek vigorously 10 times for at least 20 s. Only one side of the cheek was used for each swab. That is, when taking a microneedle swab from the left cheek, SK-2S should be applied only on the opposite right cheek.

When collecting a sample using a nasal microneedle swab, inside of the one nostril and its surroundings are wiped in a circular motion 3 times for a total of 6 times with both side of the swab (Supplementary Figure S5B). NFS-1 nasal swab sampling was also carried out in the opposite nostril according to the manufacturer’s instructions.

After buccal and nasal samples were collected, the swab handle was cut at the breakpoint, placed in a 1.5-mL tube, and stored in the refrigerator (Supplementary Figure S5A,B). Blood samples were collected using a 5-mL Streck tube and stored at freezer before the experiments.

#### 2.3.4 DNA extraction, concentration, and purity measurement

The collected buccal, nasal swabs and blood samples were subjected to a DNA extraction process using a HiGene™ gDNA Prep kit (Biofact, Daejeon, Korea) according to the manufacturer’s protocol. The experiment was conducted in two ways depending on the sample.

1) Buccal and nasal swab samples were processed as described below.

The swab was put into the tube, 450 uL of GD1 Buffer was added, and the solution was mixed by vortexing for 1 min. After vortexing, the solution was immediately transferred to a new 1.5-mL tube to prevent cell sedimentation. After 5uL of Proteinase K (20 mg/mL) and 2uL of RNase A (4 mg/mL) was dispensed, the solution was mixed by vortexing for 1 min and incubated at 56°C for 10 min. After incubation, 200uL of GD2 Buffer was added, vortexed for 10 s, and incubated at 70°C for 10 min. After incubation, centrifugation was performed at 13,000 rpm for 5 min. The supernatant of the centrifuged sample was transferred to a new 1.5-mL tube and inverted 20 times by adding 200 uL of GB buffer. After 200ul of Help B Buffer was added to a spin column equipped with a collection tube, the solution was centrifuged at 7,000 rpm for 1 min. The sample solution containing GB buffer was added to the spin column and centrifuged at 7,000 rpm for 1 min 500 uL of 80% ethanol was added to the spin column and then centrifuged at 13,000 rpm for 30 s. This process was repeated one more time. After the washing process, the spin column was idling at 13,000 rpm for 3 min. The spin column was inserted into a new 1.5-mL tube, and 50 uL of DNA Hydration Solution was added for DNA elution and incubated at room temperature for 1 min. The spin column was removed after centrifugation at 13,000 rpm for 2 min. Finally, DNA is obtained in the 1.5 mL tube for use in downstream applications.

2) Blood samples were processed as described below.

Whole blood (200ul), GD2 buffer (400 uL), and Proteinase K (20 uL, 20 mg/mL) were added into the tube and mixed by vortexing for 1 min. After incubation at 56°C for 10 min, 200 uL of GB buffer was added and inverted 20 times. Help B Buffer (200 ul) was added to the spin column equipped with a collection tube, and the column was centrifuged at 7000 rpm for 1 min. The sample solution containing GB buffer was added to the spin column. The column was centrifuged at 7,000 rpm for 1 min. The rest of the steps are the same as the process described above for buccal and nasal swab samples.

The concentration and purity of the extracted DNA samples were measured at a wavelength of 260 nm using a NanoDrop^®^ ND-1000 spectrophotometer (Nanodrop Technologies Inc., NC, United States).

#### 2.3.5 Genotyping

Genotyping was performed with 22 DNA samples, which satisfied the purity criteria (1.6–2.1). Five SNPs (rs1065757, rs3752752, rs921115, rs1009480, rs1009480, rs1820795) were randomly selected among the SNPs with a minor allele frequency in the range of approximately 0.4–0.5 in Koreans. Since SNPs are biallelic, genotyping can be more definitive when selecting SNPs among evenly distributed alleles. Genotyping was performed using the Sanger sequencing method (ABI 3500XL Sequencer^®^, Applied Biosystems, United States).

#### 2.3.6 Visual Analogue Scale (VAS)

The pain caused by the buccal and nasal microneedle swab was investigated using the Visual Analogue Scale (VAS) for the 25 subjects who participated in the clinical trial. The VAS pain index was expressed as a number from 1 (*no pain at all*) to 10 (*very painful*), and the values were interpreted through a table (Supplementary Figure S6) (Wewers and Lowe, 1990; Caffarel-Salvador et al., 2021). Bleeding was checked when the buccal and nasal microneedle swab was used, and satisfaction was expressed as a number from 1 (*very uncomfortable*) to 10 (*very satisfied*).

### 2.4 Statistical analysis

For statistical analysis of microneedle swabs and commercial swabs, we set a effect size (*f* = 0.4) (Cohen, 2013), power 80% and an alpha error probability of 0.05. Under these conditions, the total sample size needed was 66, and the sample size for each group was 22.

DNA concentrations from buccal and nasal microneedle swabs and commercial swabs were tested by paired *t*-test. The test was performed at the significance level of 0.05 (95% confidence level), and *p* ≤ .05 was considered significant.

The five SNPs were selected to compare the genotypes of DNA obtained from buccal and nasal microneedle swabs and those obtained from blood. The concordance rate (%) of genotypes from each person’s blood and buccal or nasal microneedle swab and Kappa statistics were calculated. Kappa statistics evaluate the degree of concordance between measurement methods, and it is a statistical method that measures whether the results from the two methods coincide with each other by chance.

Concordance rates and kappa statistics were calculated using only genotypes called simultaneously by both methods. If missing data occurred at any locus of the five SNP loci in the blood sample, the locus was excluded from calculation. When missing data occurred at any locus of the five SNP loci in a buccal or nasal swab sample, the locus was considered a genotype mismatch. This is because it is considered that the sampling performance through the swab has degraded.

The lower the kappa value, the lower the degree of concordance, and the closer the value is to 1, the higher the degree of agreement. Kappa values were interpreted according to the criteria (Supplementary Figure S7) (McHugh, 2012).

## 3 Results

### 3.1 Characteristics of microneedle swab

**FIGURE 1:**
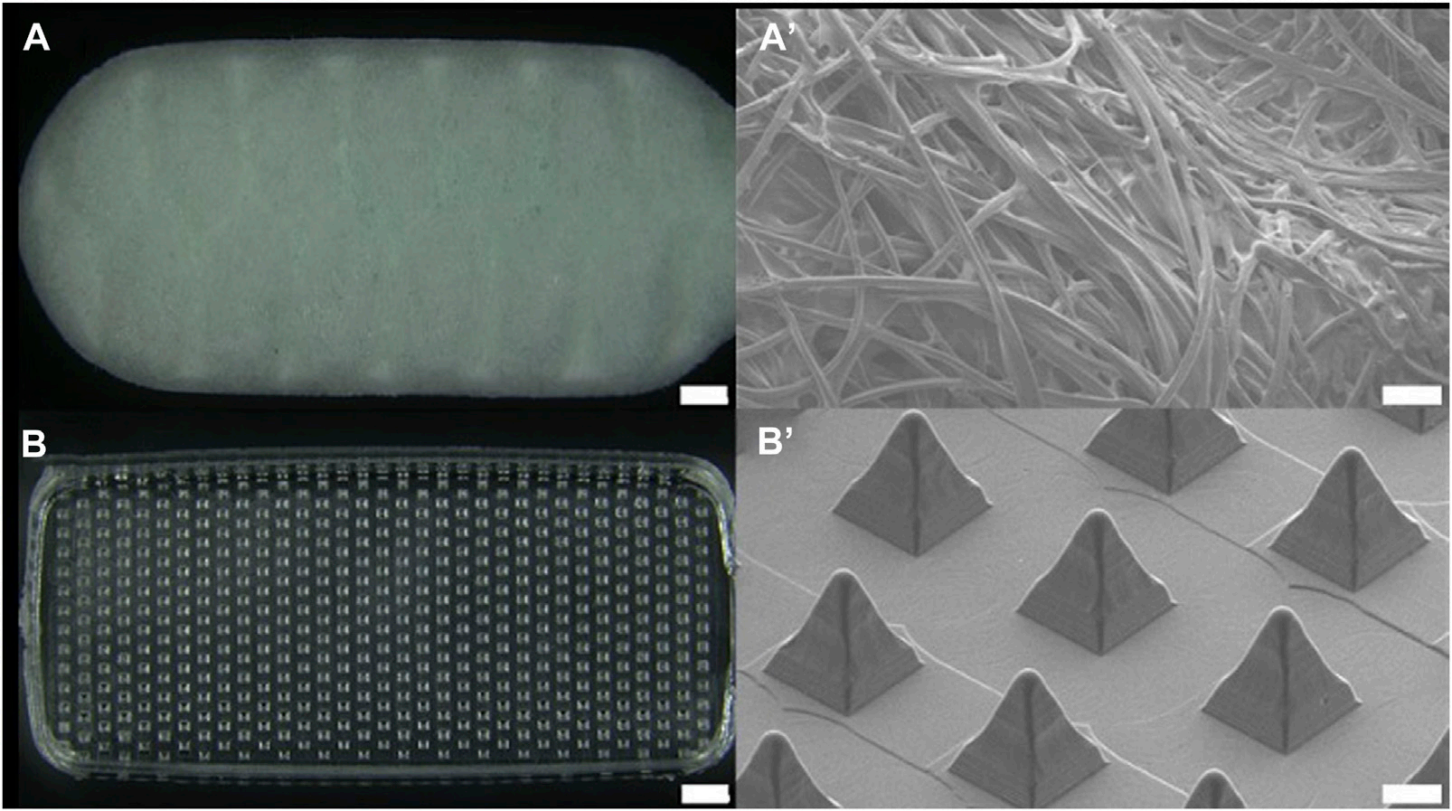
Optical image of **(A)** buccal commercial swab head (SK-2S, Isohelix, scale bar 1 mm) and **(B)** buccal microneedle swab head. Scanning electron microscopic images of **(A’)** commercial swab and **(B’)** microneedle swab (scale bar 100 μm).

**FIGURE 2:**
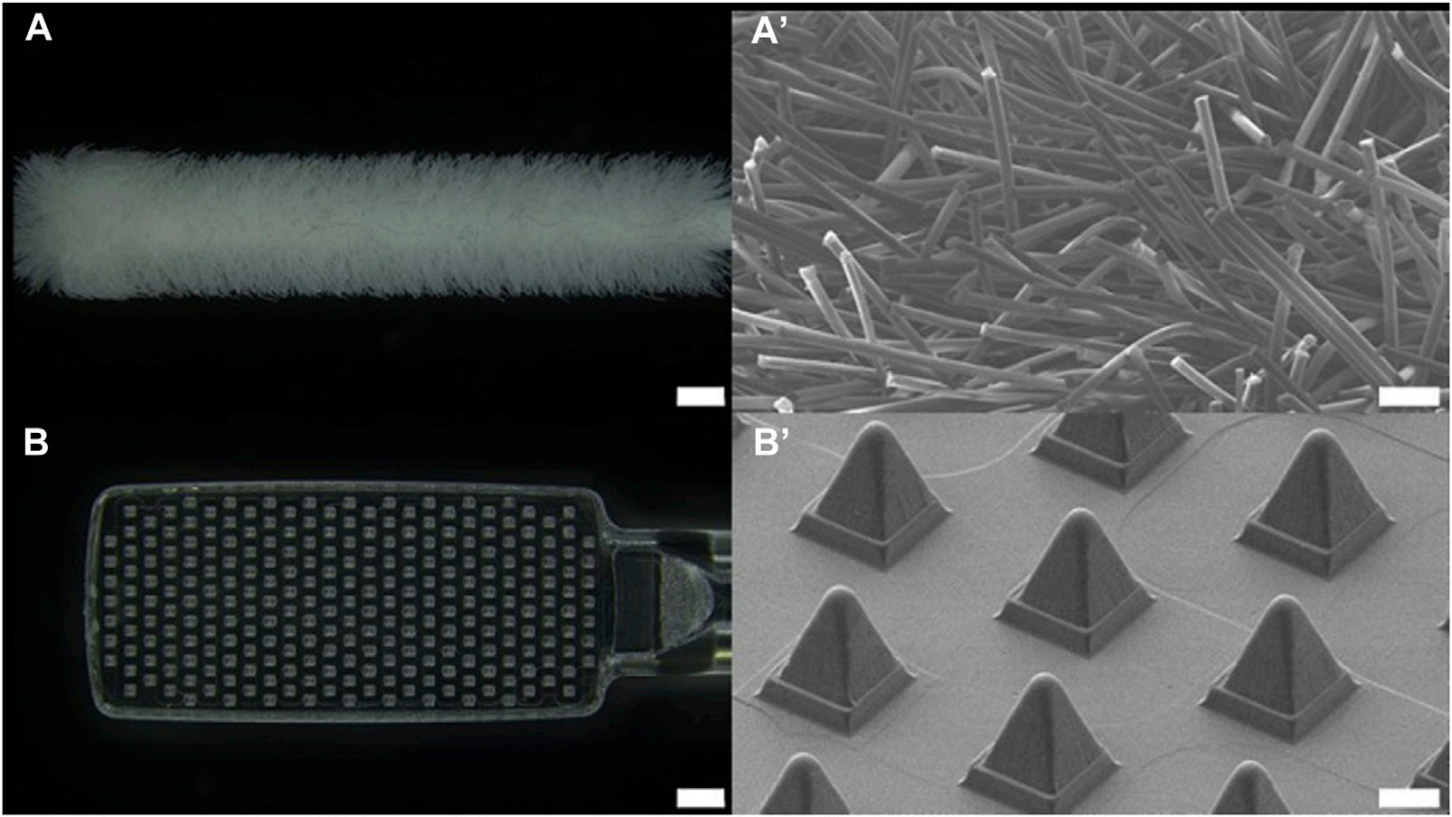
Optical image of **(A)** nasal commercial swab head (NFS-1, Noblebio, scale bar 1 mm) and **(B)** nasal microneedle swab head. Scanning electron microscopic images of **(A’)** commercial swab and **(B’)** microneedle swab (scale bar 100 μm).

The dimensions of the head of the buccal microneedle swab used in clinical trials were 6 mm × 15 mm × 2 mm (W × L × T), and the size of the head of the nasal microneedle swab was 5 mm × 10 mm × 2 mm (W × L × T). The size of the microneedle swab head was determined based on the size of the commercial buccal and nasal swabs (Figures 1, 2). One thousand samples were prepared in a single process, and when the deviation in the length of the microneedles (*n* = 100) was measured, the relative standard deviation (RSD) among the samples was less than 1%. For the buccal microneedle swab, the accessibility of the swab to the buccal mucosa was considered. The head size of the of nasal microneedle swab and the commercial swab was determined by the fact that the location of the nasal sample was 2-3 cm from the entrance of the human nose.

Considering user convenience and potential error in use, microneedles were placed on both sides of the swab head. Because the length of the microneedles is 250 μm, it is not easy to find the side where the microneedles are located. Therefore, microneedles were placed on both sides of the swab head so that the user could obtain a reproducible amount of sample from both sides with same number of repetitions of swabbing.

### 3.2 Safety of microneedle swab

#### 3.2.1 Cytotoxicity test

Cells were treated with the eluate of the polymer microneedle swab made of cyclic olefin copolymer (COC). A uniform monolayer of cells was formed during culture, but mild inhibition of cell proliferation (10% of total cells) was observed. The solvent control and negative control showed no toxicity (Grade 0) to the cultured cells, and the positive control caused cytotoxicity (Grade 4) to 75% of the total cell. Since the color of the culture medium did not change, the eluate from the microneedle swab did not change acidity. Therefore, there was very weak cytotoxicity to mouse fibroblasts. The microneedle swab showed Grade 1 (slight) cytotoxicity according to the cytotoxicity criteria of ISO 10993-5, and meets the cytotoxicity test criteria for medical devices that must be Grade 2 (mild) cytotoxicity or lower (Table 2).

**TABLE 2:** Qualitative analysis results of cytotoxicity test. Grade 0: none, Grade 1: slight, Grade 2: mild, Grade 3: moderate, Grade 4: severe.

Well	Confluent monolayer	% Growth inhibition	%Cells without intracellular granulation	% Rounding	% lysis	Reactivity	Grade
Test 1	(+)	10	0	0	0	Slight	1
Test 2	(+)	10	0	0	0	Slight	1
Test 3	(+)	10	0	0	0	Slight	1
N.C. 1	(+)	0	0	0	0	None	0
N.C. 2	(+)	0	0	0	0	None	0
N.C. 3	(+)	0	0	0	0	None	0
R.C. 1	(+)	0	0	0	0	None	0
R.C. 2	(+)	0	0	0	0	None	0
R.C. 3	(+)	0	0	0	0	None	0
P.C. 1	(−)	100	N/A	N/A	100	Severe	4
P.C. 2	(−)	100	N/A	N/A	100	Severe	4
P.C. 3	(−)	100	N/A	N/A	100	Severe	4

*Note*. Test = microneedle swab extraction; N.C., negative control; R.C., reagent control; P.C., positive control. (+) = present, (−) = absent, N/A = not applicable.

#### 3.2.2 Skin sensitivity test

A skin sensitivity test was conducted for 24 days. During the experimental period, the mortality rate due to test substance administration was 0% in polar (G2) solvent test and non-polar (G4) solvent test groups, and no general symptoms appeared in test groups (Supplementary Figure S8A). When skin reactions were observed at 24 h and 48 h after the challenge, skin reactions such as erythema and edema were not observed in the test group compared to the control group. In both G2 and G4 solvent test groups, the skin response score and sensitization rate were 0.0% and 0.0%. In both the polar (G1) and non-polar (G3) solvent control groups, the skin response score and sensitization rate were 0.0% and 0.0%, respectively, indicating that there was no solvent sensitization. As a positive control test using dinitrochlorobenzene (DNCB) according to ISO 1993-10, skin reactions such as erythema and edema were observed in the same manner. The skin response scores were calculated as 1.4, 1.7 and the sensitization rates were 100% (Supplementary Figure S8B). In addition, test groups G2 and G4 and control groups G1 and G3 showed no weight loss (Supplementary Figure S9). Therefore, the microneedle swab does not cause skin irritation, and it is made of a material that does not cause skin sensitization.

#### 3.2.3 Skin irritation test

For 3 days after administration, general symptoms and weight loss were not observed in all rabbits, and no rabbits died (Table 3). The difference between the test substance and the control substance was calculated as 0.00 for both sterile saline and cottonseed oil extracts (Supplementary Figure S10A,B). As a result of a positive control test using 0.5% SDS according to ISO 10993-23, well-defined to severe (dark red) erythema (grade 2–4) was observed at 24, 48, and 72 h after intradermal administration. Finally, the difference between the test substance and the positive control substance was 6.22, and it is a value greater than 1.0 (Supplementary Figure S10C). In the skin irritation test, each eluate from the microneedle swab satisfied the safety requirements because the difference in scores between the negative control substance and the sample is less than 1.0. Thus, the material constituting the microneedle swab was proven to be safe.

**TABLE 3:** Skin irritation test results of clinical signs and mortality for eluent from microneedle swab.

Number of rabbits	Day(s) after application				Mortality (%)
	0	1	2	3	
n = 3	Normal	Normal	Normal	Normal	0

### 3.3 Clinical study of microneedle swab

#### 3.3.1 Sterilization efficacy of microneedle swab

The result of the sterilization validation test of the microneedle swab showed that a dose of at least 15 kGy was allowed as a regular sterilization dose according to the ISO 11137 VD_max_^15^ method for the sterilization assurance level. The maximum permissible dose for the product was properly investigated through the gamma irradiation process of Greenpia Technology Co., Ltd., and the effect on product performance or packaging was confirmed to be suitable by the manufacturer.

#### 3.3.2 DNA yield and purity

The clinical research was conducted with randomly recruited 25 people from Kangbuk Samsung Hospital. The study consisted of healthy subjects between the ages of 19 and 80 years with the informed consent of all them (Table 4).

**TABLE 4:** Characteristics of the study participants.

Subject characteristics		Count	Percentage(%)
Gender	Male	18	72
	Female	7	28
Age (years)	19–25	13	52
	26–30	9	36
	60–70	3	12
Total participation		25	100

**FIGURE 3:**
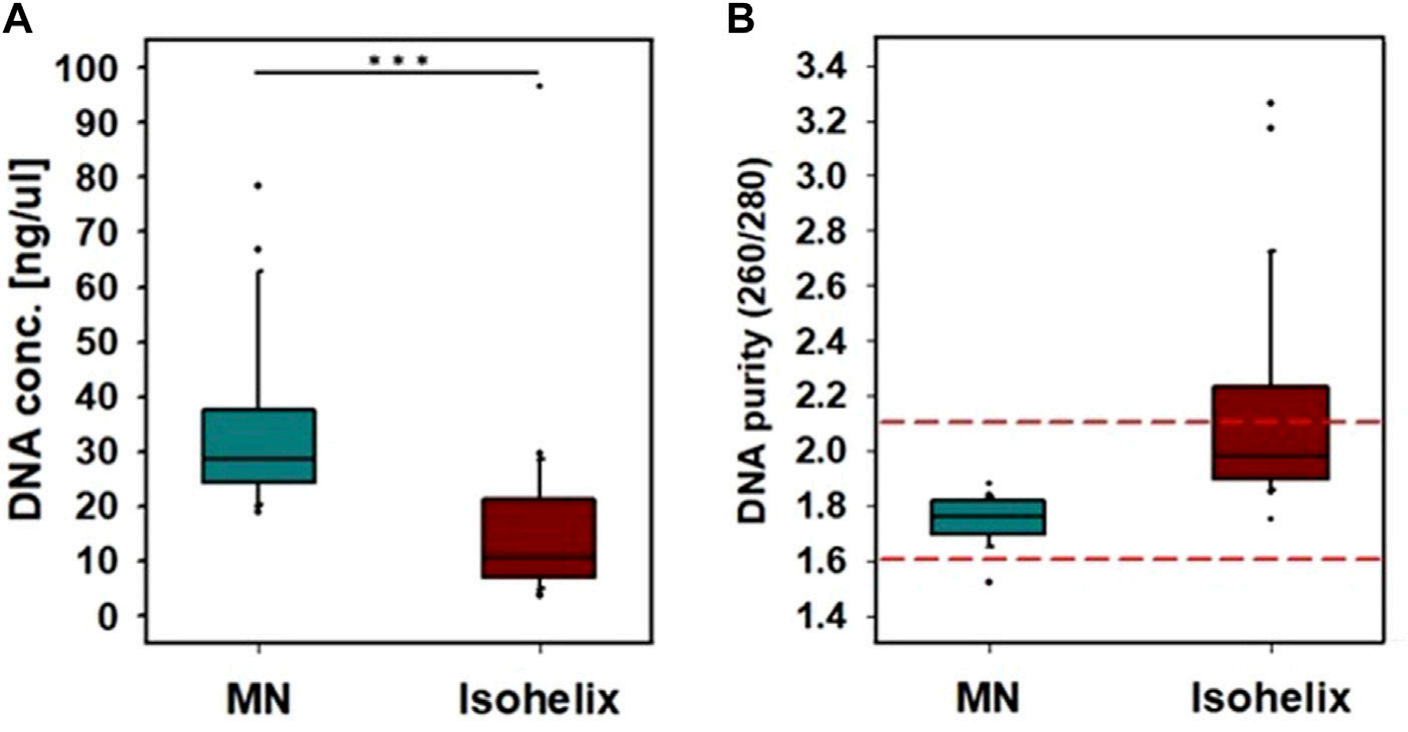
Comparison of buccal microneedle swab with Isohelix buccal swab. A sample was collected by rubbing each side of the swab 5 times for a total of 10 times wiping both sides of the microneedle swab. **(A)** DNA concentration, **(B)** DNA purity. (***: *p* < 0.001).

The amount of DNA obtained from a buccal microneedle swab was 33 ± 14 ng/μL, and the amount of DNA obtained with a commercial buccal swab (SK-2S, Isohelix) was 16 ± 18 ng/μL. Thus the buccal microneedle swab retrieved twice the amount of DNA as the commercial swab (*p* < .001) (Figure 3A). The DNA purity of the buccal microneedle swab was 1.75 ± 0.08, and the DNA purity of the commercial swab was 2.12 ± 0.37. Thus the DNA purity of the sample obtained by the buccal microneedle swab was within the appropriate range (1.6–2.1) for genetic analysis (Figure 3B) (Ahmed et al., 2013).

**FIGURE 4:**
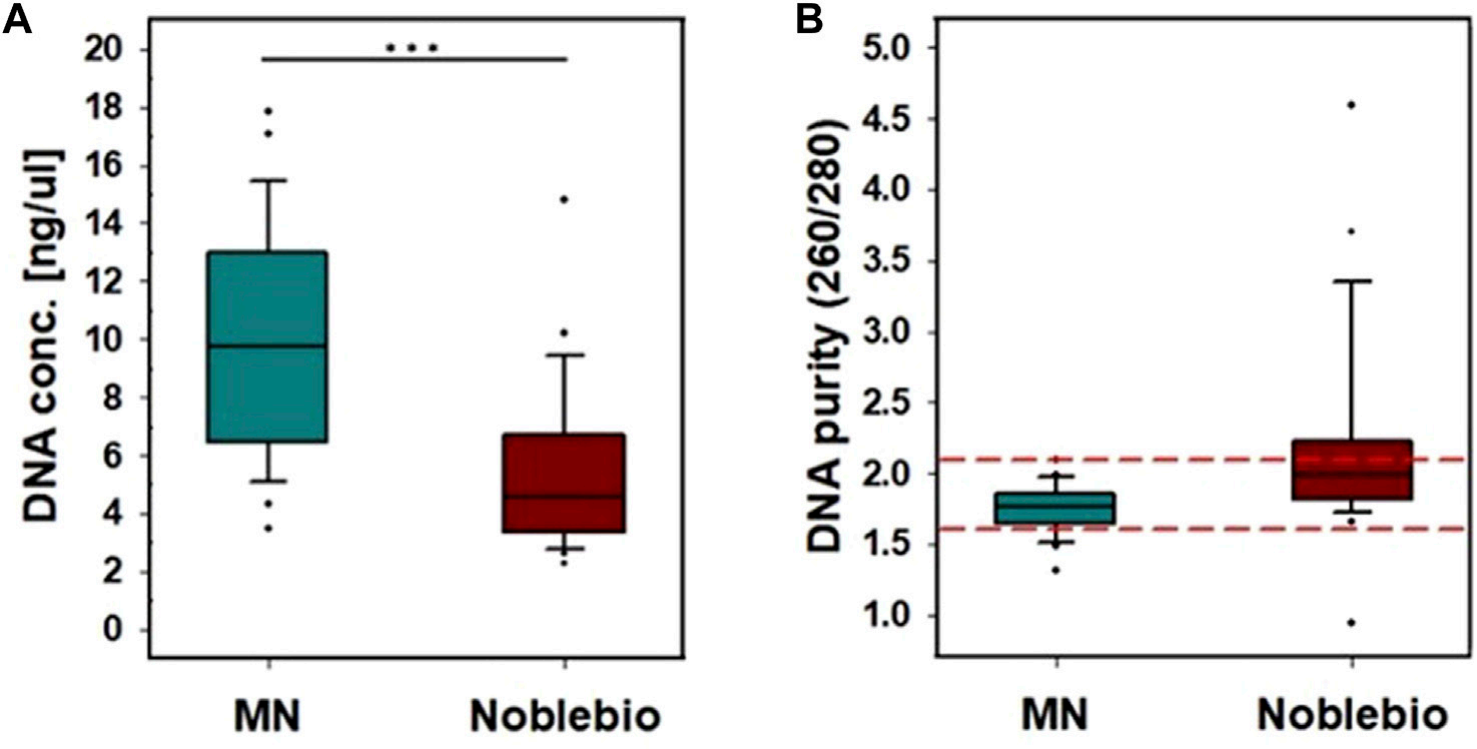
Comparison of nasal microneedle swab with Noblebio nasal swab. The inside of one nostril and its surroundings were wiped in a circular motion 3 times for a total of 6 times wiping both sides of the microneedle swab. **(A)** DNA concentration obtained, **(B)** DNA purity. (***: *p* < 0.001).

The amount of DNA obtained from the nasal cavity by the nasal microneedle swab was 9 ± 3 ng/μL, and the amount of DNA obtained by the commercial nasal swab (NFS-1, Noblebio) was 5 ± 2 ng/μL. Therefore, the amount of DNA obtained by the nasal microneedle swab was two times greater than that by the commercially available nasal swab (Figure 4A). The amount of DNA obtained is affected by how many times a swab is wiped and the pressure used when swabbing (Kim et al., 2022). The deviation in DNA amount can be caused by individual differences in the swabs used to collect samples, as shown in the variance in DNA amount in Figure 4A. The DNA purity was 1.75 ± 0.17 for the nasal microneedle swab and 2.16 ± 0.71 for NFS-1 (Noblebio), indicating that the purity of microneedle swabs was within the appropriate range (1.6–2.1) for genetic analysis (Figure 4B).

In the *ex-vivo* porcine buccal tissue experiment in our previous study, the microneedle swab showed twice as much DNA yield as the commercial rayon swab and the nylon flocked swab, and in the *in-vivo* experiment with pigs, the DNA yield of the microneedle swab was greater than that of the nylon flocked swab (Kim et al., 2022). Similar to the previous results, in the clinical trials of this study, microneedle swabs showed higher DNA yields than the commercial cotton swabs in both the buccal and the nasal samples, and the microneedle swabs showed a purity suitable for downstream genomic analysis.

#### 3.3.3 Genotype concordance

Genotype concordance was assessed with DNA samples, satisfying the DNA concentration and purity criteria. Genotypes were compared at five SNP loci from the blood of the same person, buccal, nasal microneedle swabs, and commercial swabs. The buccal and nasal microneedle swab showed a Kappa value of 1.0 for all five SNP genotypes, and the concordance rate was 100%. The SK-2S swabs (Isohelix) also showed a Kappa value of 1.0 and a concordance rate of 100%. Only one sample collected from a commercial nasal swab (NFS-1, Noblebio) in the nasal cavity showed a mismatched genotype for one SNP (rs921115) between the blood and the nasal swab, which resulted in a concordance rate of 95% and a Kappa value of 0.9 for commercial cotton swab (Table 5).

**TABLE 5:** Kappa statistics and genotype concordance rate.

Sampling	Buccal				Nasal			
Comparison target	Blood - MN (n = 22)		Blood - Isohelix (n = 22)		Blood - MN (n = 22)		Blood - Noblebio (n = 22)	
SNP	Kappa	Concordance rate (%)	Kappa	Concordance rate (%)	Kappa	Concordance rate (%)	Kappa	Concordance rate (%)
rs1065757	1.0	100	1.0	100	1.0	100	1.0	100
rs3752752	1.0	100	1.0	100	1.0	100	1.0	100
rs921115	1.0	100	1.0	100	1.0	100	0.9	95
rs1009480	1.0	100	1.0	100	1.0	100	1.0	100
rs1820795	1.0	100	1.0	100	1.0	100	1.0	100

^a^ Kappa: Kappa result ranges from 0 to 1. The higher the value of Kappa, the stronger the agreement.

This observation suggests that both the commercial buccal swabs and the buccal or nasal microneedle swabs in this study are suitable for use in downstream genetic analysis. Even though one discrepant genotype was found, the commercial nasal swabs may also be considered suitable for analysis. This discrepancy may be caused by weak frictional strength during nasal swabs, depending on the participant.

For accurate genetic analysis, a sufficient amount of DNA should be obtained and it must have sufficient purity. Commercial cotton swabs pick up fragments of epithelial cells present in saliva. When the buccal tissue is swabbed, bacteria or food present on the mucosal surface and saliva affect sample collection and analysis (Brownlow et al., 2012). In addition, cotton swabs showed low extraction efficiency and low recovery efficiency of less than 50% even though they had high absorption capacity in sample collection (Bruijns et al., 2018). Compared to conventional rayon swabs and nylon flocked swabs, microneedle swabs showed improved release efficiency because microneedles were exposed to the extraction medium (Wise et al., 2021; Kim et al., 2022). Therefore, the microneedle swab has advantages not only in sample collection but also in analysis.

#### 3.3.4 VAS measurement of pain caused by microneedle swabs

**FIGURE 5:**
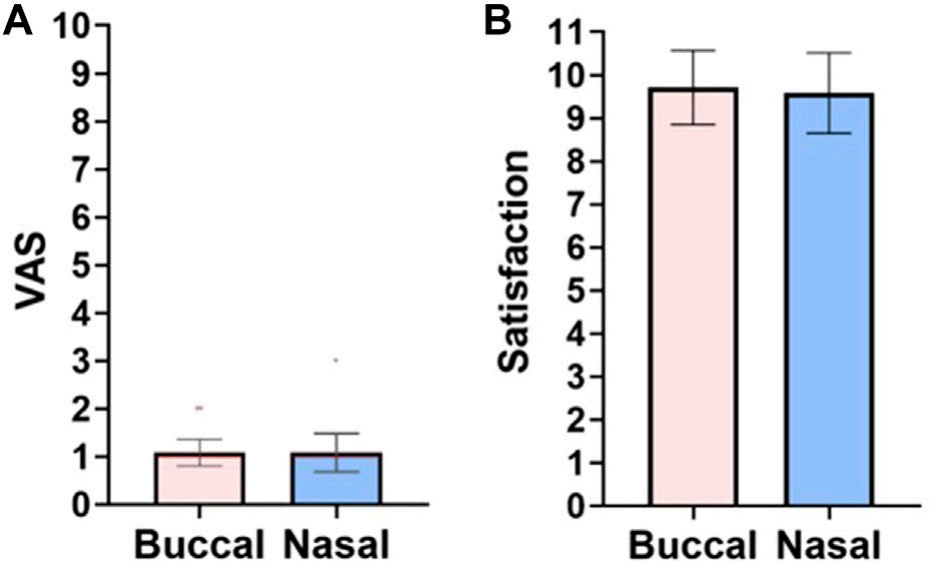
Survey results. **(A)** VAS pain scale: 0, no pain; 10, pain as bad as it could possibly be. **(B)** Satisfaction scores for buccal and nasal microneedle swabs: 0, very uncomfortable; 10, very satisfied. Pink: buccal; blue: nasal.

Since microneedle swabs contact with the buccal surface, pain caused by the swabs is a major consideration. The VAS pain scale of the buccal microneedle swab was 1.0 ± 0.2 and the nasal microneedle swab was 1.0 ± 0.4 (Figure 5A). A value of VAS 1 is very low pain perception (Supplementary Figure S6; scale of VAS pain scale). Also, none of the participants in the clinical study reported any bleeding and pain during the swabbing process.

As the microneedle length increases, pain increases proportionally (Gill et al., 2008; Jeong et al., 2017). In an *ex-vivo* experiment in a previous study, a microneedle swab with a length of 250 μm caused a scratch of about 20 μm (Kim et al., 2022). In other clinical studies, when 250 μm long microneedles pierced the skin, the pain was too low to feel (Griffin et al., 2017; Fernando et al., 2018; Samant et al., 2020). For a microneedle swab, since only the surface of the buccal mucosa is swabbed, a scratch 20 μm deep is much less than the insertion depth of a 250 μm long microneedle. As the length of the microneedle increases, more sample can be obtained, but the risk of pain or infection can also increase. Thus, the 250 μm long microneedle has an optimal geometry for obtaining samples from the mucosal surface safely and efficaciously (Kaushik et al., 2001; Gupta et al., 2011; Jeong et al., 2017).

When study participants were asked about the satisfaction and convenience of the microneedle swab, the average satisfaction reported for the buccal swab and the nasal swab was 9.7 ± 0.8 points and 9.5 ± 0.9 points (out of 10 points), respectively (Figure 5B). This means that buccal and nasal microneedle swabs are easy to use and have high accessibility because they are non-invasive and thus have excellent user accessibility.

## 4 Discussion

Buccal and nasal microneedle swabs have the advantage of obtaining a large amount of high-purity cells with less force than the previous sampling method using a cotton swab. Thus, using buccal or nasal swabs is expected to increase the accuracy of the genomic analysis. None of the participants in the clinical study reported any bleeding or pain during the swabbing process. However, there is a risk of irritation to the mucous membrane if the user does not follow the sampling guidelines. In the future, we will continue to evaluate the safety of microneedle swabs in a wider range of areas and suggest directions for use through additional clinical studies.

As we go through the COVID-19 pandemic era, the medical community is gradually moving toward a non–face-to-face testing system. Therefore, in the future, rather than visiting a hospital to collect blood and test for disease, it is expected that there will be a method of collecting samples at home using a diagnostic kit and sending the sample to be tested for disease. In addition, for large-scale genomic analysis, such as the study of population genetics, sampling methods using buccal or nasal swabs will facilitate the recruitment of many participants. In other words, individuals can easily participate in large-scale research by filling out a consent form online and collecting samples using a sampling kit delivered to their home without having to visit a hospital.

In this study, we examined the applicability of buccal and nasal microneedle swabs by comparing their performance (DNA concentration and purity) and analyzed genotype concordance with commercial swabs in a limited number of participants. In addition, safety tests (e.g., cytotoxicity test, skin sensitivity test, skin irritation test) were conducted based on guidelines established by the Korean Minister of Food and Drug Safety. Moreover, buccal and nasal microneedle swabs met the safety requirements for clinical study and commercial use. Finally, the microneedle swab for clinical study was registered as a Grade 2 medical device by the Korean Minister of Food and Drug Safety.

However, additional research is needed to apply buccal and nasal microneedle swabs in large-scale genomic analysis. That is, the reproducibility of these swabs must be evaluated under various conditions (e.g., between samplers, between sampling times, etc.). In addition, a large number of samples should be used to compare the genetic analysis performance of buccal and nasal microneedle swab samples with existing sampling methods and to verify their reliability. We plan to pursue this research in the future.

Genetic data obtained through buccal or nasal microneedle swabs is very sensitive personal information. The data contain a variety of information about an individual’s health, ancestry, and potential predisposition to various diseases. Therefore, it is important to keep these data secure to prevent unauthorized access and misuse. In addition, when a company or organization uses an individual’s genetic data for research, participants must fully understand the implications of sharing their genetic data and give prior consent for use of the data. The company or organization is also obligated to protect personal information by anonymizing such information, and the data must be used ethically.

To harness the full potential of genomics in long-term research and medical practice, it is essential to continue advancing technology and computational tools for genomics, ensuring the privacy and ethical use of genetic data, and integrating genomic data into routine medical care. In that respect, continual improvement of buccal and nasal microneedle swab sampling techniques is important to secure reliable genetic data. For example, it is necessary to evaluate how reproducible genotypes obtained based on samples collected at different times for various genetic loci for the same person are obtained. In addition, it is necessary to evaluate whether a sufficient amount of DNA is consistently obtained with high purity and whether genotyping results are error-free when using the buccal or nasal microneedle swab sampling kit in a test group consisting of a larger number of people.

## 5 Conclusion

This study was conducted to confirm the safety of the microneedle swab and to evaluate its clinical performance. The microneedle swab manufactured in good manufacturing practice (GMP) satisfied all safety test standards regarding cytotoxicity, skin sensitivity, and skin irritation. It also passed the sterilization efficacy test. Buccal and nasal microneedle swabs showed two times higher DNA yields and greater purity than commercial cotton swabs. When the genotypes of SNPs were compared between samples collected by microneedle swabs and blood samples, all genotypes tested were concordant, confirming that microneedle swabs were suitable for clinical use. The VAS pain index during sampling was 1, which is difficult to perceive, and user convenience also showed satisfactory characteristics.

For large-scale genomic analysis in areas such as population genetics, buccal or nasal microneedle swab sampling methods allow for easy recruitment of many participants. Although this study evaluated the performance and safety of the buccal or nasal microneedle swab using a small number of samples, such evaluations should be conducted with a larger number of samples in the future.

Therefore, the newly developed microneedle swab can effectively and easily collect DNA from the buccal and nasal mucosa. Microneedle swabs can be used for DTC tests as well as disease diagnosis and prevention through genomic analysis, so its utilization is expected to be high.

## Data Availability

The datasets presented in this study can be found in online repositories. The names of the repository/repositories and accession number(s) can be found in the article/Supplementary Material.
