# Supplementary material for: Safety tests and clinical research on buccal and nasal microneedle swabs for genomic analysis
Source: Front Bioeng Biotechnol. 2023 Dec 5;11:1296832. doi: 10.3389/fbioe.2023.1296832 (PMC10729317; doi:10.3389/fbioe.2023.1296832)
Supplement: Supplementary file 1 [file DataSheet1.docx]

**Supporting information**

**Safety tests and clinical research on buccal and nasal microneedle swabs for genomic analysis**

JeongHyeon Kim^a^, Gil-Hwan Sung^b^, Seung-Ki Baek^b^, Won-Jun Jo^b^, Jae-Woo Moon^c^, Gyeong Ryeong Kim^c^, Wonsub Gim^c^, Jung Ho Park^d^, Hae-Jin Hu^c,*^, Jung-Hwan Park ^a,*^

^a^ Department of Bionano Technology and Gachon BioNano Research Institute, Gachon University, Gyeonggi-do, Republic of Korea

^b^ QuadMedicine R&D Centre, QuadMedicine Co., Ltd, Seongnam, Republic of Korea

^c^ Endomics, Inc. Seongnam-si, Gyeonggi-do, Republic of Korea

^d^ Department of Medicine, Kangbuk Samsung Hospital, Sungkyunkwan University School of Medicine, Seoul 03181, Republic of Korea

***** Authors to whom correspondence should be addressed:

Hae-Jin Hu (haejinhu@goendomics.com)

Jung-Hwan Park (pa90201@gachon.ac.kr)

**
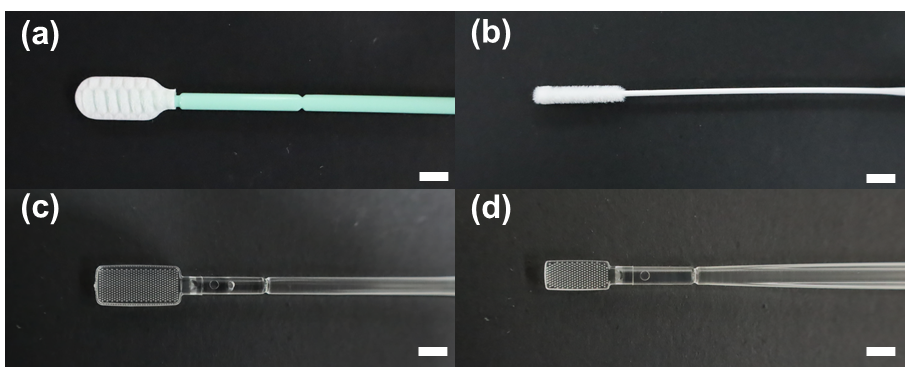
**

**Figure S1.** Optical images of (a) commercial buccal swab: rayon swab from Isohelix; (b) commercial nasal swab: flocked nylon swabs from Noblebio; (c)buccal microneedle swab; (d) nasal microneedle swab (scale bar 1 cm). The head of the microneedle swab was separated at the breakpoint (dashed line) to put head in solution


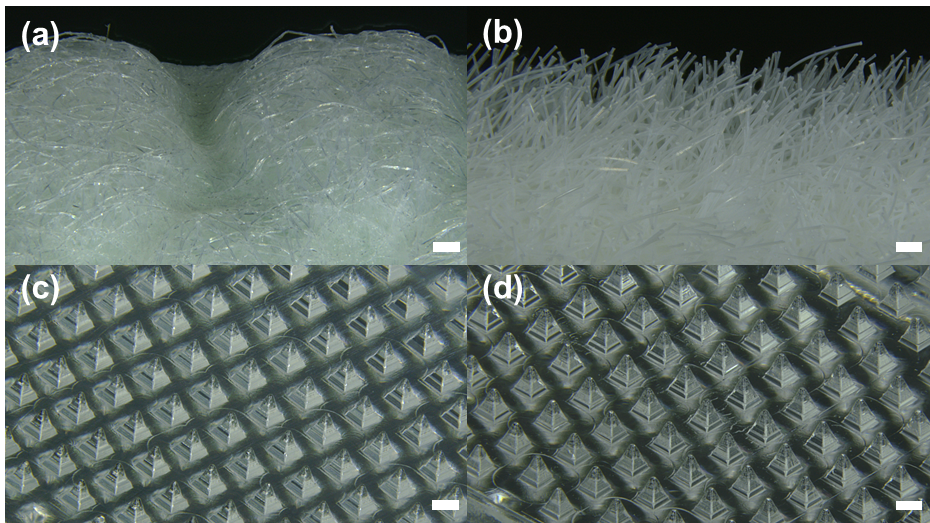


**Figure S2.** Optical image of swab surface. (a) Commercial buccal swab: rayon swab from Isohelix; (b) commercial nasal swab: flocked nylon swab from Noblebio; (c) buccal microneedle swab; (d) nasal microneedle swab (scale bar 200 μm).

**
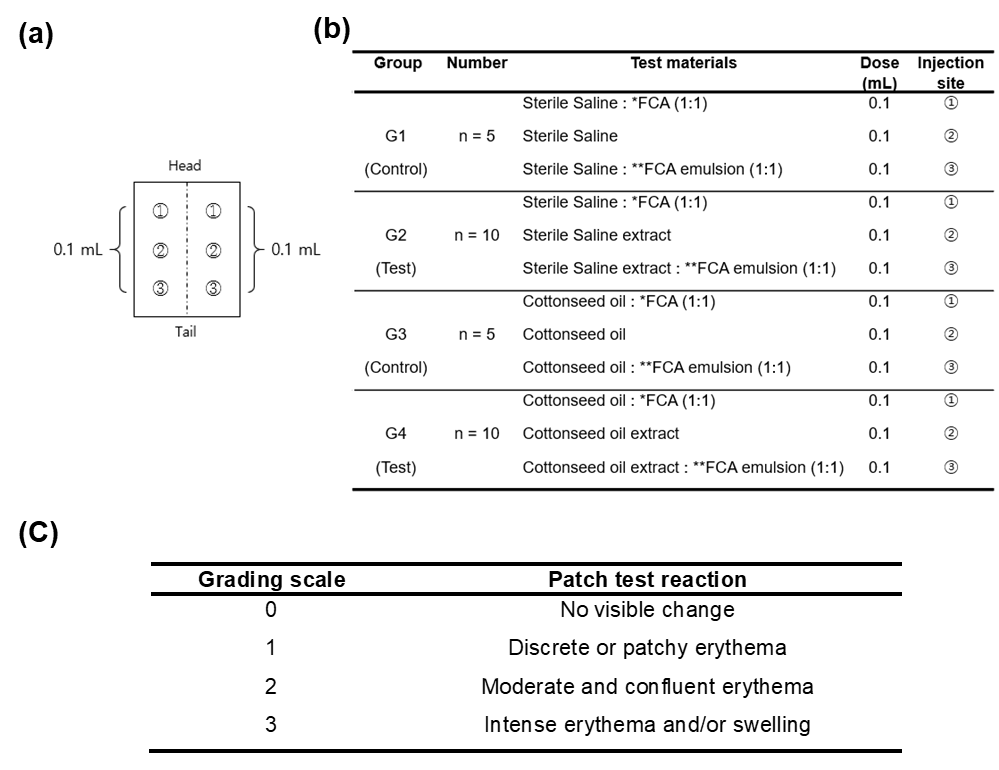
**

**Figure S3.** Skin sensitivity test. (a) Intradermal injection site of guinea pig. (b) Intradermal injection solution by group. (c) Magnusson and Kligman scale for skin response. *FCA: Freund’s Complete Adjuvant, **FCA emulsion (vehicle [1:1]).


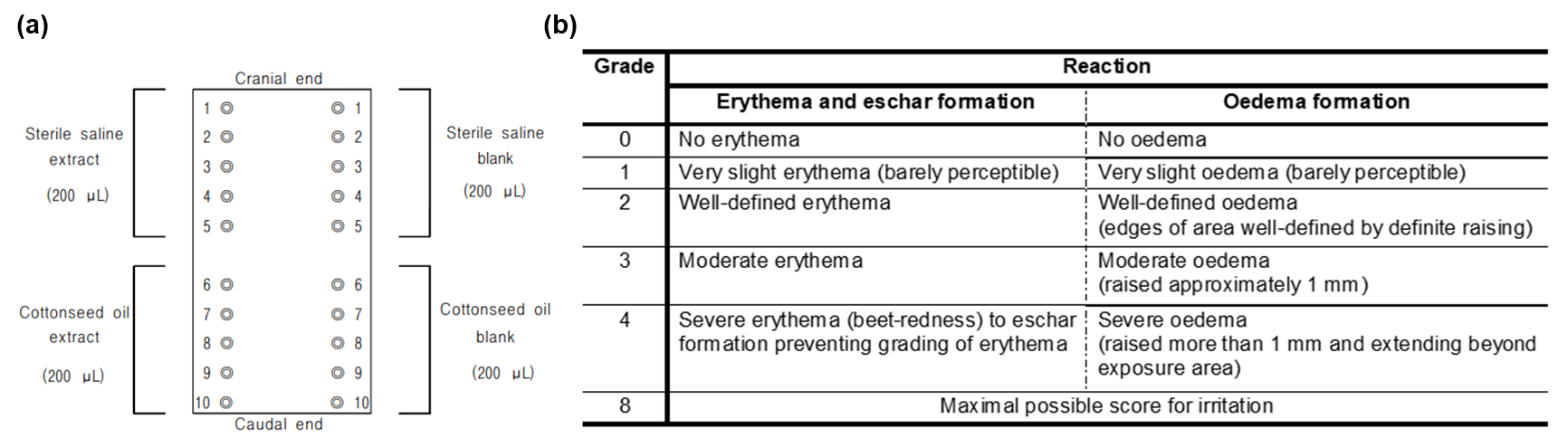


**Figure S4.** Skin irritation test. (a) Intradermal injection site of rabbit (b) Grade for skin irritation test. ISO 10993-23 : 2021, Tests for irritation.

(a)

| **Instructions for use of Buccal Microneedle Swab** | |
| --- | --- |
| **1. Open microneedle swab**  Take out the microneedle swab from the wrapper by pulling the end of wrapper. | 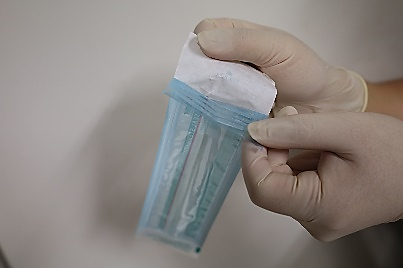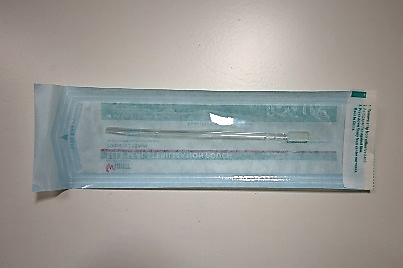 |
| **2. Swab buccal mucosa**   - Gently insert the entire head of microneedle swab into the mouth until you feel contact on buccal mucus. - Swab in a back and forth motion by rubbing each side of the swab 5 times and wiping both sides 10 times in total for at least 20 seconds. | 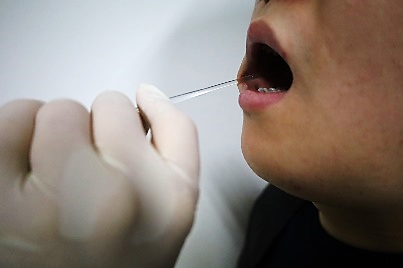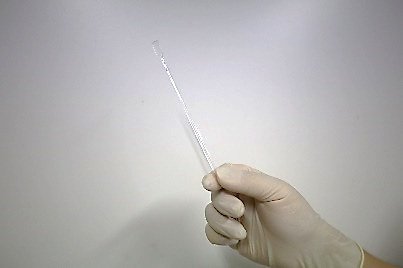 |
| **3. Put swab in tube**   - Break the swab by bending the breakpoint of microneedle swab. - Dip the swab into the prepared tube and close. | 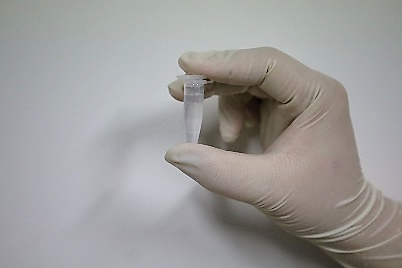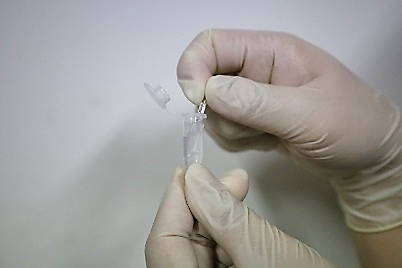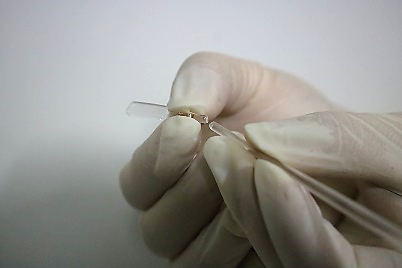 |

(b)

| **Instructions for use of Nasal Microneedle Swab** | |
| --- | --- |
| **1. Open microneedle swab**  Take out the microneedle swab from the wrapper by pulling the end of wrapper. | 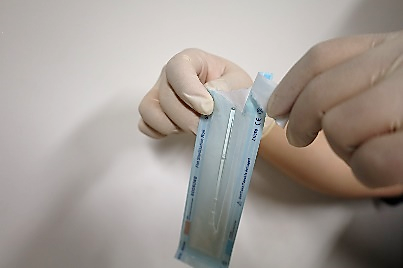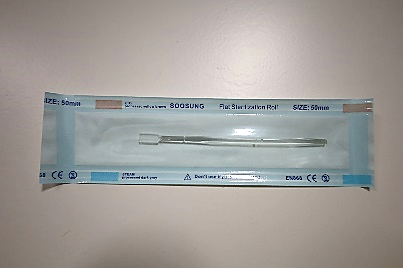 |
| **2. Swab nasal mucosa**   - Gently insert the entire head of microneedle swab into the nostril about 2-3 cm or until you feel contact on nasal mucus. - Swab nostril surroundings in a circular motion 3 times for a total of 6 times. | 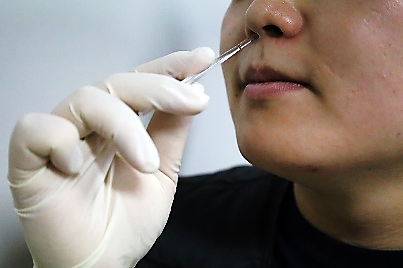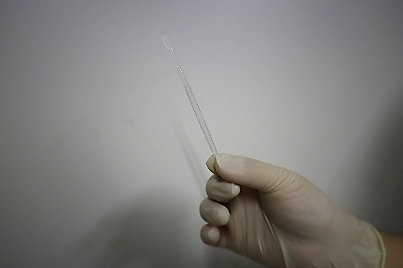 |
| **3. Put swab in tube**   - Break the swab by bending the breakpoint of microneedle swab. - Dip the swab into the prepared tube and close. | 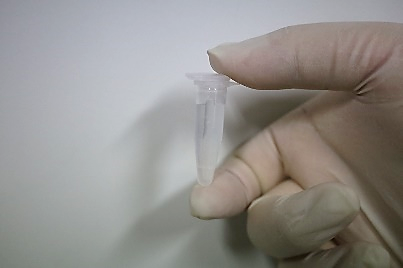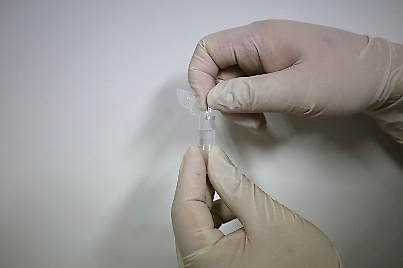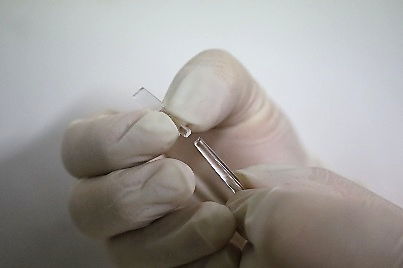 |

**Figure S5.** Instruction for use of (a) buccal microneedle swab and (b) nasal microneedle swab for collection of sample.


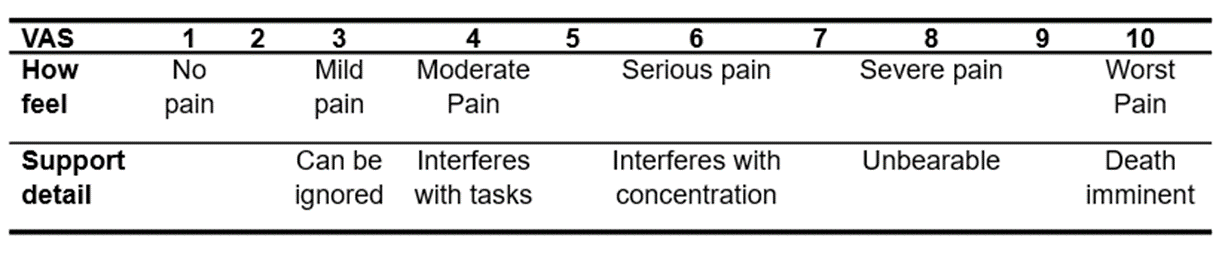


**Figure S6.** Scale of VAS pain score.


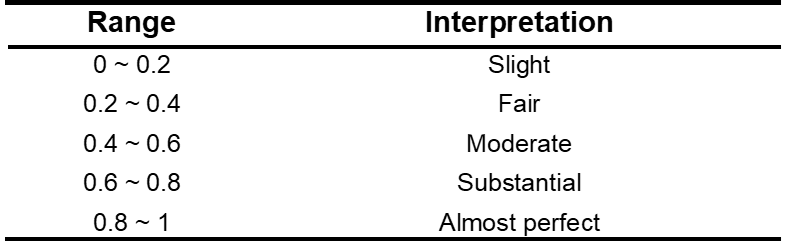


**Figure S7.** Interpretation of Kappa statistic.


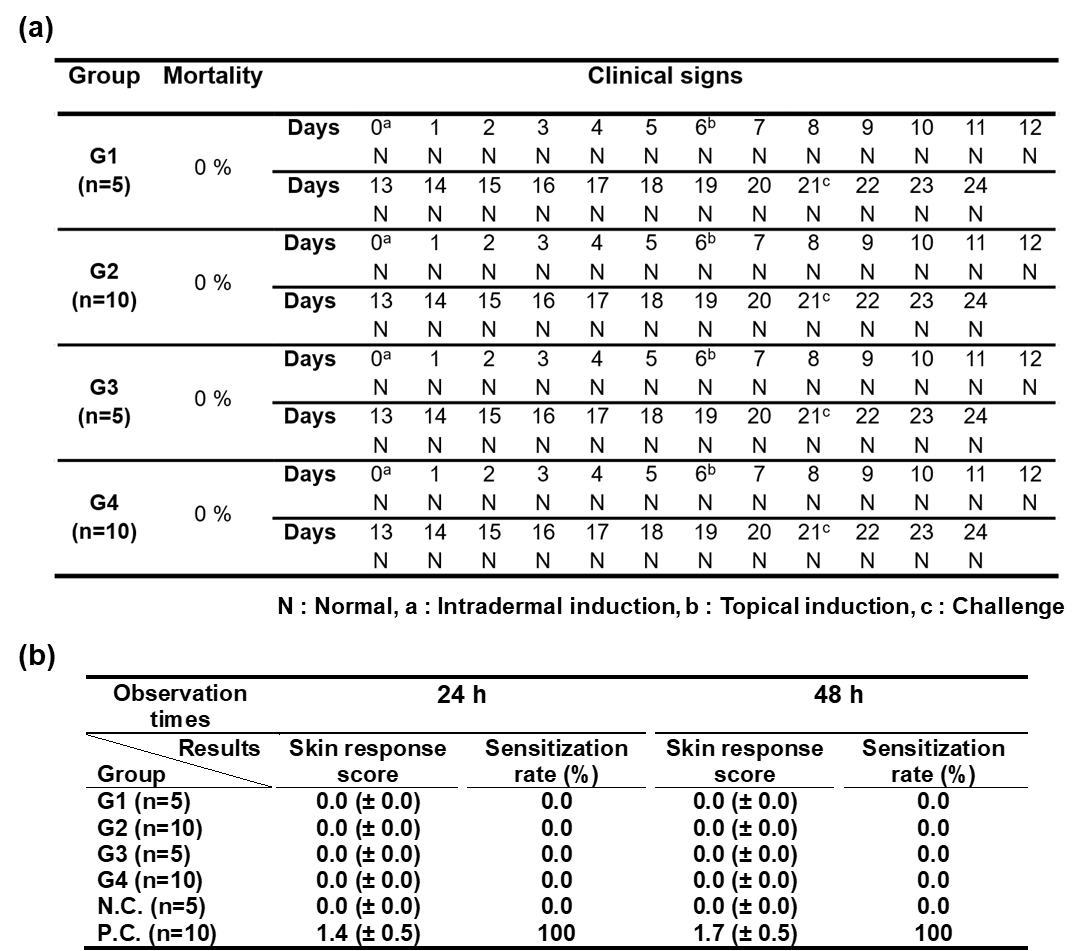


**Figure S8.** Results of skin sensitivity test. (a) Mortality and clinical signs. (b) Evaluation of the skin response. (Skin response mean value [± *SD*], N.C.: negative control, P.C. positive control).


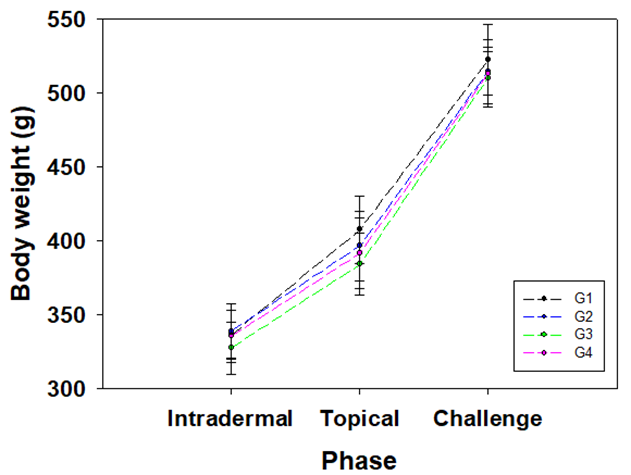


**Figure S9.** Change of body weight of mouse during skin sensitivity test.

(a)

| **Skin irritation test of saline extracts** | | | | | | | | | | | | | |
| --- | --- | --- | --- | --- | --- | --- | --- | --- | --- | --- | --- | --- | --- |
| Animal number | | Test | | | | | | Control | | | | | |
|  |  | 1101 | | 1102 | | 1103 | | 1101 | | 1102 | | 1103 | |
| Ob. ^1)^ time | Injection site No. | ER ^2)^ | OE ^3)^ | ER | OE | ER | OE | ER | OE | ER | OE | ER | OE |
|  | 1 | 0 | 0 | 0 | 0 | 0 | 0 | 0 | 0 | 0 | 0 | 0 | 0 |
|  | 2 | 0 | 0 | 0 | 0 | 0 | 0 | 0 | 0 | 0 | 0 | 0 | 0 |
| 24 h ^4)^ | 3 | 0 | 0 | 0 | 0 | 0 | 0 | 0 | 0 | 0 | 0 | 0 | 0 |
|  | 4 | 0 | 0 | 0 | 0 | 0 | 0 | 0 | 0 | 0 | 0 | 0 | 0 |
|  | 5 | 0 | 0 | 0 | 0 | 0 | 0 | 0 | 0 | 0 | 0 | 0 | 0 |
|  | 1 | 0 | 0 | 0 | 0 | 0 | 0 | 0 | 0 | 0 | 0 | 0 | 0 |
|  | 2 | 0 | 0 | 0 | 0 | 0 | 0 | 0 | 0 | 0 | 0 | 0 | 0 |
| 48 h | 3 | 0 | 0 | 0 | 0 | 0 | 0 | 0 | 0 | 0 | 0 | 0 | 0 |
|  | 4 | 0 | 0 | 0 | 0 | 0 | 0 | 0 | 0 | 0 | 0 | 0 | 0 |
|  | 5 | 0 | 0 | 0 | 0 | 0 | 0 | 0 | 0 | 0 | 0 | 0 | 0 |
|  | 1 | 0 | 0 | 0 | 0 | 0 | 0 | 0 | 0 | 0 | 0 | 0 | 0 |
|  | 2 | 0 | 0 | 0 | 0 | 0 | 0 | 0 | 0 | 0 | 0 | 0 | 0 |
| 72 h | 3 | 0 | 0 | 0 | 0 | 0 | 0 | 0 | 0 | 0 | 0 | 0 | 0 |
|  | 4 | 0 | 0 | 0 | 0 | 0 | 0 | 0 | 0 | 0 | 0 | 0 | 0 |
|  | 5 | 0 | 0 | 0 | 0 | 0 | 0 | 0 | 0 | 0 | 0 | 0 | 0 |
| Sum | | 0 | 0 | 0 | 0 | 0 | 0 | 0 | 0 | 0 | 0 | 0 | 0 |
| Sum / 15* | | 0.00 | | 0.00 | | 0.00 | | 0.00 | | 0.00 | | 0.00 | |
| Mean score / 3 ** | | 0.00 | | | | | | 0.00 | | | | | |
| Score difference*** | | 0.00 | | | | | | | | | | | |

(b)

| **Skin irritation test of cottonseed oil extracts** | | | | | | | | | | | | | |
| --- | --- | --- | --- | --- | --- | --- | --- | --- | --- | --- | --- | --- | --- |
| Animal number | | Test | | | | | | Control | | | | | |
|  |  | 1101 | | 1102 | | 1103 | | 1101 | | 1102 | | 1103 | |
| Ob. ^1)^ time | Injection site No. | ER ^2)^ | OE ^3)^ | ER | OE | ER | OE | ER | OE | ER | OE | ER | OE |
|  | 1 | 1 | 0 | 1 | 0 | 1 | 0 | 1 | 0 | 1 | 0 | 1 | 0 |
|  | 2 | 1 | 0 | 1 | 0 | 1 | 0 | 1 | 0 | 1 | 0 | 1 | 0 |
| 24 h ^4)^ | 3 | 1 | 0 | 1 | 0 | 1 | 0 | 1 | 0 | 1 | 0 | 1 | 0 |
|  | 4 | 1 | 0 | 1 | 0 | 1 | 0 | 1 | 0 | 1 | 0 | 1 | 0 |
|  | 5 | 1 | 0 | 1 | 0 | 1 | 0 | 1 | 0 | 1 | 0 | 1 | 0 |
|  | 1 | 1 | 0 | 1 | 0 | 1 | 0 | 1 | 0 | 1 | 0 | 1 | 0 |
|  | 2 | 1 | 0 | 1 | 0 | 1 | 0 | 1 | 0 | 1 | 0 | 1 | 0 |
| 48 h | 3 | 1 | 0 | 1 | 0 | 1 | 0 | 1 | 0 | 1 | 0 | 1 | 0 |
|  | 4 | 1 | 0 | 1 | 0 | 1 | 0 | 1 | 0 | 1 | 0 | 1 | 0 |
|  | 5 | 1 | 0 | 1 | 0 | 1 | 0 | 1 | 0 | 1 | 0 | 1 | 0 |
|  | 1 | 1 | 0 | 1 | 0 | 1 | 0 | 1 | 0 | 1 | 0 | 1 | 0 |
|  | 2 | 1 | 0 | 1 | 0 | 1 | 0 | 1 | 0 | 1 | 0 | 1 | 0 |
| 72 h | 3 | 1 | 0 | 1 | 0 | 1 | 0 | 1 | 0 | 1 | 0 | 1 | 0 |
|  | 4 | 1 | 0 | 1 | 0 | 1 | 0 | 1 | 0 | 1 | 0 | 1 | 0 |
|  | 5 | 1 | 0 | 1 | 0 | 1 | 0 | 1 | 0 | 1 | 0 | 1 | 0 |
| Sum | | 15 | 0 | 15 | 0 | 15 | 0 | 15 | 0 | 15 | 0 | 15 | 0 |
| Sum / 15* | | 1.00 | | 1.00 | | 1.00 | | 1.00 | | 1.00 | | 1.00 | |
| Mean score / 3 ** | | 1.00 | | | | | | 1.00 | | | | | |
| Score difference*** | | 0.00 | | | | | | | | | | | |

(c)

| **Skin irritation test of irritation reactivity** | | | | | | | | | | | | | |
| --- | --- | --- | --- | --- | --- | --- | --- | --- | --- | --- | --- | --- | --- |
| Animal number | | 1101 | | | | 1102 | | | | 1103 | | | |
|  |  | Test | | Control | | Test | | Control | | Test | | Control | |
| Ob. ^1)^ time | Injection site No. | ER ^2)^ | OE ^3)^ | ER | OE | ER | OE | ER | OE | ER | OE | ER | OE |
|  | 1 | 3 | 3 | 0 | 0 | 3 | 3 | 0 | 0 | 3 | 3 | 0 | 0 |
|  | 2 | 3 | 3 | 0 | 0 | 3 | 3 | 0 | 0 | 3 | 3 | 0 | 0 |
| 24 h ^4)^ | 3 | 3 | 3 | 0 | 0 | 3 | 3 | 0 | 0 | 3 | 3 | 0 | 0 |
|  | 4 | 3 | 3 | 0 | 0 | 3 | 3 | 0 | 0 | 3 | 3 | 0 | 0 |
|  | 5 | 3 | 3 | 0 | 0 | 3 | 3 | 0 | 0 | 3 | 3 | 0 | 0 |
|  | 1 | 3 | 3 | 0 | 0 | 3 | 3 | 0 | 0 | 3 | 3 | 0 | 0 |
|  | 2 | 3 | 3 | 0 | 0 | 3 | 3 | 0 | 0 | 3 | 3 | 0 | 0 |
| 48 h | 3 | 3 | 3 | 0 | 0 | 3 | 3 | 0 | 0 | 3 | 3 | 0 | 0 |
|  | 4 | 3 | 3 | 0 | 0 | 3 | 3 | 0 | 0 | 3 | 3 | 0 | 0 |
|  | 5 | 3 | 3 | 0 | 0 | 3 | 3 | 0 | 0 | 3 | 3 | 0 | 0 |
|  | 1 | 3 | 4 | 0 | 0 | 4 | 4 | 0 | 0 | 2 | 3 | 0 | 0 |
|  | 2 | 3 | 4 | 0 | 0 | 4 | 4 | 0 | 0 | 2 | 3 | 0 | 0 |
| 72 h | 3 | 3 | 4 | 0 | 0 | 4 | 4 | 0 | 0 | 2 | 3 | 0 | 0 |
|  | 4 | 3 | 4 | 0 | 0 | 4 | 4 | 0 | 0 | 2 | 3 | 0 | 0 |
|  | 5 | 3 | 4 | 0 | 0 | 4 | 4 | 0 | 0 | 2 | 3 | 0 | 0 |
| Sum | | 45 | 50 | 0 | 0 | 50 | 50 | 0 | 0 | 40 | 45 | 0 | 0 |
| Sum / 15* | | 6.33 | | 0.00 | | 6.67 | | 0.00 | | 5.67 | | 0.00 | |
| Mean score / 3** | | 6.33 | | | | 6.67 | | | | 5.67 | | | |
| Score difference*** | | 6.22 | | | | | | | | | | | |

**Figure S10.** Results of skin irritation test. (a) Saline extracts. (b) Cottonseed oil extracts. (c) irritation reactivity. ^1)^ Ob.: Observation, ^2)^ ER: Erythema, ^3)^ OE: Oedema, ^4)^ h: Hour, 15*: 3 scoring time points (24h, 48h, 72h test observation time) × 5 test injection sites, 3**: Number of animals, Score difference***: Treatment irritation score − Control irritation score.


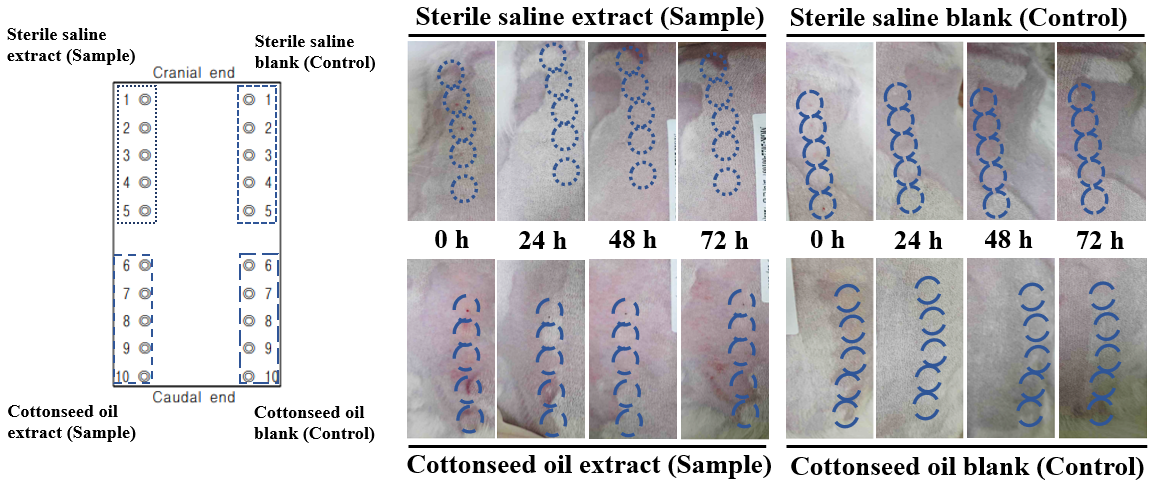


**Figure S11.** Photographs after intradermal administration for skin irritation test. Circles indicate the injection site of blank and extract from sample.
